# Anti-Inflammatory Effect of a Polyphenol-Enriched Fraction from *Acalypha wilkesiana* on Lipopolysaccharide-Stimulated RAW 264.7 Macrophages and Acetaminophen-Induced Liver Injury in Mice

**DOI:** 10.1155/2018/7858094

**Published:** 2018-08-07

**Authors:** Hongtan Wu, Haiyue Pang, Yupei Chen, Lisen Huang, Huaxin Liu, Yongbiao Zheng, Cuiling Sun, Gang Zhang, Gueyhorng Wang

**Affiliations:** ^1^Application Technique Engineering Center of Natural Cosmeceuticals, College of Fuijan Province, Xiamen Medical College, Fujian, Xiamen 361023, China; ^2^Research Center of Natural Cosmeceuticals Engineering, Xiamen Medical College, Fujian, Xiamen 361023, China; ^3^Key Laboratory of Functional and Clinical Translational Medicine, Fujian Province University, Xiamen 361023, China; ^4^Engineering Research Center of Industrial Microbiology of Ministry of Education, College of Life Sciences, Fujian Normal University, Fujian, Fuzhou 350117, China; ^5^Analysis and Measurement Center, School of Pharmaceutical Sciences, Xiamen University, Fujian, Xiamen 361001, China; ^6^Technology and Engineering Center for Marine Biomedical Resource Utilization, Xiamen Medical College, Fujian, Xiamen 361023, China

## Abstract

A polyphenol-enriched fraction (PEF) from *Acalypha wilkesiana*, whose leaves have been traditionally utilized for the treatment of diverse medical ailments, was investigated for the anti-inflammatory effect and molecular mechanisms by using lipopolysaccharide- (LPS-) stimulated RAW 264.7 macrophages and acetaminophen- (APAP-) induced liver injury mouse model. Results showed that PEF significantly attenuated LPS-induced nitric oxide (NO) and prostaglandin E_2_ (PGE_2_) production and suppressed the expression of inducible nitric oxide synthase (iNOS) and cyclooxygenase (COX-2) in RAW 264.7 macrophages. PEF also reduced the secretion of proinflammatory cytokines including tumor necrosis factor-*α* (TNF-*α*), interleukin- (IL-) 1*β*, and IL-6 in LPS-stimulated RAW 264.7 macrophages. Moreover, PEF potently inhibited LPS-induced phosphorylation of mitogen-activated protein kinases (MAPKs) as well as the activation of nuclear factor-*κ*B (NF-κB) by preventing the degradation of inhibitor κB-*α* (IκB-*α*). In vivo, PEF pretreatment ameliorated APAP-induced liver injury and hepatic inflammation, as presented by decreased hepatic damage indicators and proinflammatory factors at both plasma and gene levels. Additionally, PEF pretreatment remarkably diminished Toll-like receptor 3 (TLR3) and TLR4 expression and the subsequent MAPKs and NF-*κ*B activation. HPLC analysis revealed that two predominantly polyphenolic compounds present in PEF were geraniin and corilagin. These results indicated that PEF has an anti-inflammatory effect, and its molecular mechanisms may be involved in the inactivation of the TLR/MAPK/NF-*κ*B signaling pathway, suggesting the therapeutic potential of PEF for inflammatory diseases.

## 1. Introduction

Inflammation, which is considered to play a crucial role in the body's immune defense system, functions to eliminate the initial cause of cell injury and clear out necrotic cells and tissues damaged from the original insult and the inflammatory process [1]. However, hypernomic or abnormal inflammatory reaction and process could cause a series of diseases, for example, cardiovascular disease, neurodegeneration, and cancer [2]. Hence, it is of great importance to develop efficient agents for the treatment of inflammatory-mediated diseases.

LPS-activated macrophage serves as a well-established experimental model for evaluating the anti-inflammatory activities of various synthetic or naturally derived agents in vitro [3, 4]. Macrophages are known to play a key role in the host defense mechanism in response to infection or injury and mediate the inflammation and the immune system directly or indirectly [5]. Redundant inflammatory mediators (NO and PGE_2_) and proinflammatory cytokines (TNF-*α*, IL-1*β*, and IL-6) produced by activated macrophages have been considered crucial causes for acute inflammatory responses as well as chronic inflammatory diseases including rheumatoid arthritis, osteoarthritis, and inflammatory bowel disease [6, 7]. Gene expression of cytokines and inflammatory proteins such as iNOS and COX-2 is largely dependent on the activation of the transcription factor NF-*κ*B [8, 9]. NF-*κ*B, a protein complex that controls DNA transcription, cytokine production, and cell survival, plays a critical role in the regulation of immune responses [10]. Under unstimulated conditions, NF-*κ*B is sequestered in the cytoplasm by binding to I*κ*B-*α*. The activation of NF-*κ*B is initiated by the signal-induced phosphorylation of a kinase called the I*κ*B kinase (IKK), which in turn promotes the ubiquitin-dependent degradation of I*κ*B-*α*, resulting in the nuclear translocation of NF-*κ*B. Then, the activated NF-*κ*B binds specifically to *κ*B sites in the promoter regions of target genes and enhances the expression of inflammatory mediators and proinflammatory cytokines [11]. MAPKs, a family of serine/threonine kinases associated with the inflammatory process, are also reported to regulate the activation of NF-*κ*B and subsequent expression of inflammatory mediators and proinflammatory cytokines [12, 13]. Therefore, synthetic or naturally derived compounds that inhibit the activation of NF-*κ*B and MAPKs can be considered and developed as potential therapeutic medicines for inflammatory diseases [14].

APAP is a widely used analgesic/antipyretic drug with few adverse effects at therapeutic doses. However, at an extremely high dose, APAP causes severe hepatic necrosis and even fulminant acute liver failure. The primary mechanistic insight into the pathogenesis of APAP-induced hepatotoxicity is regarded as the excess accumulation of a highly toxic and reactive metabolite, N-acetyl-p-benzoquinone imine (NAPQI), which depletes the cellular glutathione (GSH), resulting in severe oxidative stress and centrilobular necrosis [15, 16]. While toxic metabolites of APAP account for the primary hepatic damage, accumulating evidence indicates that the activation of the liver's innate immune cells and subsequent downstream participation of inflammatory mediators and proinflammatory cytokines exacerbate the injury [17]. It is clearly demonstrated that early hepatic cell necrosis caused by toxic metabolites of APAP leads to several cell contents' leakage including heat shock proteins (HSPs), nuclear and mitochondrial DNA fragments, and others collectively referred to as damage-associated molecular patterns (DAMPs), which directly bind and activate Toll-like receptors (TLRs) on sentinel cells such as macrophages and dendritic cells. The activated sentinel cells cause the release of massive inflammatory mediators and proinflammatory cytokines and recruit monocytes and neutrophils into the liver, ultimately aggravating the initial injury and liver cell necrosis [18–20]. TLRs, a class of pattern recognition receptors that recognize structurally conserved molecules derived from microbes, play important roles in the molecular mechanisms of inflammation, particularly TLR4 [21]. TLR4 has been known to be the predominant modulator in the pathogenesis of inflammatory responses involved in drug-induced acute liver injury [22, 23]. Numerous reports have demonstrated that TLR4 regulates the inflammatory responses mainly through the stimulation of several intracellular signaling pathways, including NF-*κ*B, MAPKs, and phosphatidylinositol 3-kinase (PI3K)/protein kinase B (AKT) pathways [10, 24]. Hence, the inhibition of the TLR/MAPK/NF-*κ*B signaling pathway can be a therapeutic strategy to alleviate APAP-induced hepatotoxic effects.

In recent years, there was an increasing interest in finding natural products from plant materials especially traditional herbs which are considered to be harmless and free from toxic side effects [25]. It is generally accepted that natural products, which are abundant sources of bioactive compounds, can be developed as beneficial dietary supplements and therapeutic drug candidates [26, 27]. Polyphenolic compounds (PCs) such as phenolic acids and flavonoids have drawn increasing attention for their medicinal functions and health benefits including antioxidant and anti-inflammatory properties [28, 29]. *Acalypha wilkesiana*, which belongs to the spurge family (*Euphorbiaceae*), is a medical and ornamental plant widely distributed around the world, especially in the tropics of Africa. The leaves of *A. wilkesiana* have been employed in folk medicines for the treatment of many ailments [30, 31]. A previous phytochemical analysis indicated the presence of alkaloids, tannins, triterpenoids, sesquiterpenoids, and polyphenols in *A. wilkesiana* extract, which was shown to have analgesic, antipyretic, antibacterial, and anti-inflammatory activities [32, 33]. However, to the best of our knowledge, there is no scientific research available to describe the molecular mechanisms of its anti-inflammatory activity and what kinds of compounds it is related to.

Thus, the aim of the present study was to evaluate the anti-inflammatory effect of a polyphenol-enriched fraction from *A. wilkesiana* by using LPS-stimulated RAW 264.7 macrophages and an APAP-induced liver injury mouse model and explore the possible regulation mechanisms underlying its actions. Moreover, the main constituents of PEF were identified and quantified by reversed-phase high-performance liquid chromatography (RP-HPLC) for a better understanding of the bioactive compounds responsible for its anti-inflammatory and hepatoprotective effects.

## 2. Materials and Methods

### 2.1. Chemicals and Reagents

LPS, NG-nitro-L-arginine methyl ester (L-NAME), 3-(4,5-dimethylthiazol-2yl)-2,5-diphenyltetrazolium bromide (MTT), hematoxylin, and eosin were purchased from Sigma-Aldrich (St. Louis, MO, USA); PrimeScript™ RT Master Mix and SYBR Green Master Mix were purchased from Takara Biotechnology (Dalian, China). Oligonucleotides were synthesized by Sangon Biotech (Shanghai, China). APAP was purchased from Sangon Biotech (Shanghai, China). Assay kits of alanine aminotransferase (ALT), aspartate aminotransferase (AST), lactate dehydrogenase (LDH), and myeloperoxidase (MPO) were purchased from Nanjing Jiancheng Institute of Biotechnology (Nanjing, China). Nitric Oxide Assay Kit and Nuclear and Cytoplasmic Protein Extraction Kit were purchased from Beyotime Institute of Biotechnology (Shanghai, China). TRIzol, Dulbecco's Modified Eagle's Medium (DMED), fetal bovine serum (FBS), penicillin, and streptomycin were purchased from Invitrogen (Carlsbad, CA, USA). All other chemicals and reagents were analytical grade and commercially available.

### 2.2. Plant Material, Extraction, and Preparation Procedure

Leaves of *A. wilkesiana* were supplied by Associate Professor Zhongning Lin from the Agricultural Ecology Institute of Fujian Academy of Agricultural Sciences. The air-dried and powdered leaves of *A. wilkesiana* (700 g) were extracted with 7000 mL of deionized water at 100°C for 1 h. The decoction was filtered and then concentrated by a rotary vacuum evaporator (Eyela, Tokyo, Japan) with a water bath at 55°C to give a water extract, which was then resuspended in water and named as WE. WE was filtered again and then added to a Diaion HP-20 resin (1.3 kg, Mitsubishi Chemical, Tokyo, Japan) column (8 cm × 50 cm id), which was further washed successively with two column volumes of water and 75% aqueous ethanol to give two fractions. The water portion containing sugars, proteins, and salts was named as WF. Polyphenols were retained and then eluted with 75% aqueous ethanol to afford the EtOH fraction, designated as PEF. Each fraction was concentrated, lyophilized, and then stored at 4°C for further analysis. The yields of WF and PEF were 17.4 g and 20.4 g, respectively.

### 2.3. Determination of Total Polyphenols and Total Flavonoids

The total phenolic content was measured as micrograms of gallic acid equivalents (GAE) per milligram of extract (*μ*g GAE/mg extract) by the Folin-Ciocalteu method as mentioned previously [34]. In brief, aliquots of samples or standard solutions (0.5 mL) were mixed with 0.5 mL of Folin-Ciocalteu reagent and allowed to react for 2 min. Then, 0.5 mL of 10% sodium carbonate (Na_2_CO_3_) solution and 3.5 mL of distilled water were added and mixed. Solutions were kept in the dark for 2 h. Absorbance was measured with a microplate reader (Molecular Devices, Sunnyvale, CA) at 760 nm. The total phenolic content was calculated as GAE from a calibration curve. Data were presented as the average of triplicate analyses.

Total flavonoid content was measured as micrograms of rutin equivalents (RE) per milligram of extract (*μ*g RE/mg extract) using a modified colorimetric method as described previously [34]. Briefly, aliquots of samples or standard solutions (0.5 mL) were mixed with 0.1 mL of 5% sodium nitrite (NaNO_2_) solution. After 4 min, 0.1 mL of 10% aluminium chloride (AlCl_3_) solution was added and allowed to react for another 4 min. Then, 0.3 mL of 4% sodium hydroxide (NaOH) solution was added to the mixture and adjusted to 5 mL with 60% ethanol followed by a thorough mixing. The reaction was kept for 10 min. Absorbance was determined at 510 nm. Total flavonoid content was calculated as RE according to a calibration curve. Data were presented as the average of triplicate analyses.

### 2.4. Measurement of NO, PGE_2_, TNF-*α*, IL-1*β*, and IL-6

RAW 264.7 macrophages were cultured in 96-well plates at an initial density of 2 × 10^4^ cells/well to adhere overnight and incubated with the samples (WE, WF, or PEF) ranging from 10 to 200 *μ*g/mL or L-NAME (1 mM) for 2 h and then stimulated with or without LPS (1 *μ*g/mL) for 24 h; the doses were selected according to previous studies of the effects of plant extracts on LPS-stimulated RAW 264.7 macrophages [4, 35–37]. The cultured medium was collected, centrifugated and then stored at −80°C until tested. The nitrite concentration in the cultured medium was measured as an indicator of NO production, determined by the Nitric Oxide Assay Kit (Beyotime Institute of Biotechnology, Shanghai, China) according to the manufacturer's instructions. Briefly, 50 *μ*L of cultured medium was mixed with 100 *μ*L of Griess reagent, and then, the nitrite concentration was measured at 540 nm from a standard curve derived from NaNO_2_. The level of PGE_2_ in the cultured medium was quantitated by the commercially enzyme-linked immunosorbent assay (ELISA) kit (R&D Systems, Minneapolis, MN), and levels of TNF-*α*, IL-1*β*, and IL-6 in the cultured medium were quantitated by the ELISA kits (ABclonal Biotechnology, Wuhan, China) according to the manufacturer's instructions.

### 2.5. Cell Culture and Viability Assay

RAW 264.7 macrophages were purchased from the Shanghai Cell Bank of the Chinese Academy of Sciences (Shanghai, China). Cells were cultured in DMEM supplemented with 10% FBS, 100 U/mL penicillin, and 100 *μ*g/mL streptomycin at 37°C in a humidified incubator with 5% CO_2_-enriched atmosphere. Cell viability was determined by MTT assay [38]. In brief, cells were seeded into 96-well plates at an initial density of 2 × 10^4^ cells/well to adhere overnight and incubated with the samples (WE, WF, or PEF) ranging from 10 to 200 *μ*g/mL or L-NAME (1 mM) for 2 h and then stimulated with or without LPS (1 *μ*g/mL) for 24 h. Then, 10 *μ*L of 5 mg/mL MTT was added into each well, followed by an additional 4 h incubation. The cultured medium was removed, and the formazan crystals were dissolved in 150 *μ*L DMSO. Absorbance of formazan solution was measured at 490 nm. Cell viability was expressed as a relative percentage of the untreated control cells.

### 2.6. Preparation of Cytosolic and Nuclear Extracts

RAW 264.7 macrophages were seeded at an initial density of 5 × 10^6^ cells in each 60 mm dish and incubated overnight. Cells were pretreated with PEF (10, 100, and 200 *μ*g/mL) for 2 h and then stimulated with or without LPS (1 *μ*g/mL) for 0.5 h. Before extraction, cells were harvested and washed once with ice-cold PBS buffer, and then, cytosolic and nuclear extracts were prepared by using the Nuclear and Cytoplasmic Protein Extraction Kit (Beyotime Institute of Biotechnology, Shanghai, China) according to the manufacturer's instructions.

### 2.7. Animals and Experimental Design

A total of 40 male C57BL/6 mice, 6–8 weeks old, were purchased from the Laboratory Animal Center, Xiamen University (Xiamen, China). Mice were maintained with free access to water and food in the animal room with a constant temperature of 22 ± 2°C and a 12 h light-dark cycle. The use of animals and experimental methods was in compliance with the National Institutes of Health Guidelines for the Care and Use of Laboratory Animals. All efforts were made to minimize the suffering and reduced the use of animals. Mice were randomly divided into 5 groups (8 mice per group). PEF was administered intragastrically at 100, 200, and 400 mg/kg body weight once daily for 7 consecutive days; the doses were selected based on previous studies of the effects of plant extracts on drug-induced liver injury mouse models [25, 39–41]. The mice from the control and the APAP-intoxicated groups were also given the same volume of the vehicle for 7 consecutive days. Two hours after the final administration, the mice in the APAP-intoxicated group and PEF-protective group were administrated intragastrically with a single dose of 500 mg/kg body weight APAP, and the mice in the control group were given a single of the vehicle [34]. Mice were sacrificed for collecting blood samples and liver tissues after 6 h APAP treatment.

Blood samples were centrifugated at 10,000 ×g at 4°C for serum collection and then kept at −80°C until being used for the bioassays. Liver tissues were immediately collected, placed in ice-cold PBS buffer, and then cut into pieces. One-halves were fixed by 4% paraformaldehyde for histopathological analysis, and the other halves were flash-frozen in liquid nitrogen and stored at −80°C for further analysis.

### 2.8. Measurement of Biochemical Parameters in Serum

Serum activities of ALT, AST, LDH, and MPO were measured by using the corresponding commercial kits (Nanjing Jiancheng Institute of Biotechnology, Nanjing, China) according to the manufacturer's instructions. Serum TNF-*α*, IL-1*β*, and IL-6 were quantitated by the ELISA kits (ABclonal Biotechnology, Wuhan, China) according to the manufacturer's instructions.

### 2.9. Histopathological Analysis

Liver tissues were fixed in 4% paraformaldehyde, dehydrated, and then embedded in molten paraffin [25]. Sections of 5 *μ*m thickness were taken and stained with hematoxylin and eosin (H&E) to monitor histological changes by microscopy.

### 2.10. Total RNA Extraction, Reverse Transcription, and Real-Time Quantitative PCR Analysis

Total RNA was extracted from cells or liver tissues using TRIzol reagent (Invitrogen, Carlsbad, CA, USA), and then, 1 *μ*g of total RNA was used for reverse transcription by using PrimeScript RT Master Mix (Takara Biotechnology, Dalian, China) according to the manufacturer's instructions. Real-time quantitative PCR analysis was performed by the Roche LightCycler® 480 System (Roche Group, Switzerland) using SYBR Green Master Mix (Takara Biotechnology, Dalian, China) referring to the protocol. PCR primers used for specific genes were listed as follows: iNOS: (forward 5′-CAACCAGTATTATGGCTCCT-3′ and reverse 5′-GTGACAGCCCGGTCTTTCCA-3′), COX-2: (forward 5′-CAGCAAATCCTTGCTGTTCC-3′ and reverse 5′-TGGGCAAAGAATGCAAACATC-3′), TNF-*α*: (forward 5′-AGCCGATGGGTTGTACCTTG-3′ and reverse 5′-ATAGCAAATCGGCTGACGGT-3′), IL-1*β*: (forward 5′-TCCAGGATGAGGACATGAGCAC-3′ and reverse 5′-GAACGTCACACACCAGCAGGTTA-3′), IL-6: (forward 5′-GAGTGGCTAAGGACCAAGACC-3′ and reverse 5′-AACGCACTAGGTTTGCCGA-3′), MCP-1: (forward 5′-ATGCAGTTAACGCCCCACTC-3′ and reverse 5′-CCCATTCCTTCTTGGGGTCA-3′), MIP-1*α*: (forward 5′-TGAATGCCTGAGAGTCTTGG-3′ and reverse 5′-TTGGCAGCAAACAGCTTATC-3′), TLR3: (forward 5′-CCTCCAACTGTCTACCAGTTCC-3′ and reverse 5′-GCCTGGCTAAGTTATTGTGC-3′), TLR4: (forward 5′-GGTGTGAAATTGAGACAATTGA-3′ and reverse 5′-GTTTCCTGTCAGTACCAAGGTTG-3′), and GAPDH: (forward 5′-AAACGGCTACCACATCCAAG-3′ and reverse 5′-CCTCCAATGGATCCTCGTTA-3′). Thermocycler conditions included an initial denaturation at 94°C for 3 min, 40 cycles of denaturation at 94°C for 30 sec, annealing at 60°C for 30 sec, and extension at 72°C for 30 sec, followed by a 2 min extension at 72°C. GAPDH was used as an internal control for normalization. Relative mRNA expression levels were calculated based on the Comparative-Ct method (2^−ΔΔCt^ method).

### 2.11. Western Blot Analysis

Western blot analysis was performed as described previously [34]. Briefly, total proteins were extracted from cells or frozen liver tissues and the protein concentrations were determined. Then, aliquots of proteins (20–40 *μ*g) were separated by sodium dodecyl sulfate-polyacrylamide gel electrophoresis (SDS-PAGE) and transferred onto polyvinylidene difluoride (PVDF) membranes (Millipore Corp., Billerica, MA, USA). The membranes were blocked with 5% bovine serum albumin (BSA) in Tris-buffered saline with Tween 20 (TBST) at room temperature for 1 h and then incubated with a 1 : 1000 dilution of various primary antibodies in 5% BSA with TBST at 4°C overnight. Antibodies against iNOS (13120), IKK*α* (11930), IKK*β* (8943), phospho-IKK*α*/*β* (2697), NF-*κ*B p65 (8242), phospho-NF-*κ*B p65 (3033), I*κ*B-*α* (4814), phospho-I*κ*B-*α* (2859), JNK (9252), phospho-JNK (4668), ERK1/2 (4695), phospho-ERK1/2 (4370), p38 (8690), phospho-p38 (4511), and GAPDH (2118) were purchased from Cell Signaling Technology (Beverly, MA, USA), antibodies against TLR3 (ab62566) and TLR4 (ab13556) were purchased from Abcam (Cambridge, UK), and antibody against COX-2 (A1253) was purchased from ABclonal Biotechnology (Wuhan, China). The membranes were washed three times with TBST and then incubated with horseradish peroxidase-conjugated secondary antibodies to rabbit IgG (7074) or to mouse IgG (7076) (1 : 5000 dilution for each) purchased from Jackson ImmunoResearch Laboratories (West Grove, PA, USA) at room temperature for 1 h. After three washings with TBST, the immunoreactive proteins were visualized using the SuperSignal West Pico Kit (Thermo Fisher Scientific Pierce, IL, USA) according to the manufacturer's instructions and then imaged using the ChemiDoc™ XRS+ System (Bio-Rad, CA, USA).

### 2.12. Separation, Identification, and Determination of Main Components in PEF

The lyophilized powder of PEF was redissolved in 20% methanol and then subjected to ODS (50 *μ*m, YMC Co. Ltd., Kyoto, Japan) column chromatography eluting with 20% aqueous methanol. The eluent was concentrated by a rotary vacuum evaporator with a water bath at 40°C and then purified by repeated semipreparative reversed-phase HPLC on a YMC Triart C18 column (10 × 250 mm, 5 *μ*m, YMC Co. Ltd., Kyoto, Japan) with 40% aqueous ethanol to yield compound 1 (15 mg) and compound 2 (10.8 mg), respectively. Structures of 1 and 2 were confirmed by ^1^H-NMR, ^13^C-NMR, and MS analysis. Purities of 1 and 2 were 96.2% and 94.7%, respectively, as judged by an area normalization method. Both of the isolated 1 and 2 were used as reference standards. Compounds 1 and 2 in PEF were quantified using the HPLC method. The used HPLC equipment was an Agilent 1260 series HPLC system equipped with a diode array detector, an autosampler, and an open LAB CDS chemstation workstation (Agilent, USA). The analysis was performed on an Eclipse Plus column (3.5 *μ*m, 100 mm × 4.6 mm id, Agilent Technologies, USA) using a gradient elution from 5% A (acetonitrile) to 80% B (water containing 0.1% formic acid) in 35 min at a flow rate of 1.0 mL/min. The detection was carried out at 280 nm. All separations were performed at 25°C. The lyophilized powder of PEF was dissolved in 50% methanol at a concentration of 0.61 mg/mL. All samples and mobile phases were filtered through a 0.45 *μ*m membrane filter (Millipore) and then degassed in an ultrasonic bath prior to use. Identifications of 1 and 2 on the chromatograph of PEF were performed by comparing their retention times (*t*_R_) with those of standards identified. The linear regression equation for each calibration curve was established by plotting the amount of each standard compound injected against the average peak area. Quantitative data of 1 and 2 were calculated from their respective standard curves.

### 2.13. Statistical Analysis

Results are expressed as the mean ± standard deviation (SD), and all assays were performed at least in triplicate. The statistical analysis was conducted by using SPSS 20.0 software (SPSS, Chicago, IL, USA). All values were tested for normal distribution and equal variance. When homogeneous variances were confirmed, data were analyzed by means of one-way analysis of variance (ANOVA), followed by Turkey-Kramer's test for post hoc analysis. Other statistical evaluations were performed by Student's *t*-test. Differences were considered significant of *p* < 0.05.

## 3. Results

### 3.1. Total Phenolic and Total Flavonoid Contents in Different *A. wilkesiana* Extracts

Total phenolic contents of WE, WF, and PEF were determined by using the Folin-Ciocalteu method. As presented in Table 1, WE, WF, and PEF contained 261.1 ± 2.5, 87 ± 3.1, and 661.4 ± 5.2 *μ*g GAE/mg extract, respectively. Similar results were obtained from the determination of total flavonoid contents of WE, WF, and PEF, shown as 152.6 ± 3.8, 38 ± 3.5, and 306.5 ± 7.5 *μ*g RE/mg extract, respectively. This quantitative analysis indicated that PEF contained the highest content of total phenolics and total flavonoids, implying that PEF was a polyphenol-enriched fraction.

### 3.2. Effect of Different *A. wilkesiana* Extracts on the Production of NO

**Figure 1:**
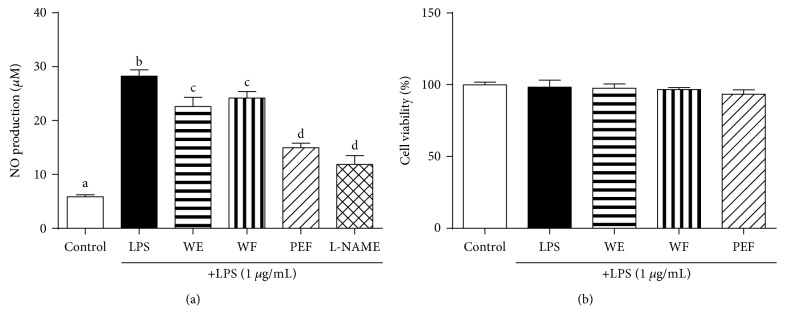
Effect of WE, WF, and PEF on LPS-stimulated NO production and cell viability in RAW 264.7 macrophages. RAW 264.7 macrophages were preincubated with 100 *μ*g/mL WE, WF, or PEF for 2 h and then treated with 1 *μ*g/mL LPS for 24 h. (a) The nitrite concentration in the cultured medium was measured as an indicator of NO production by the Griess reaction. L-NAME (1 mM), an iNOS inhibitor, was used as a positive control. (b) The cell viability was evaluated by MTT assay. Results are shown as the mean ± SD (*n* = 3). The different letters represent the statistical differences at *p* < 0.05 among the groups by Tukey-Kramer's test.

It is generally recognized that the NO-inhibitory effect is a major index to evaluate the effectiveness of anti-inflammatory agents [14, 42]. Thus, the effect of different *A. wilkesiana* extracts on LPS-stimulated NO production in RAW 264.7 macrophages was determined. L-NAME, an inhibitor of iNOS which regulates NO production, was used as a positive control. As shown in Figure 1(a), NO production, measured as nitrite concentration in the cultured medium, was significantly increased by LPS treatment compared to the untreated control cells. However, the above-mentioned alteration was prominently alleviated by PEF pretreatment as well as L-NAME pretreatment, while other tested extracts showed no significant decreases. Further cytotoxicity analysis indicated that no matter PEF, L-NAME, or other tested extracts had no effect on the viability of RAW 264.7 macrophages (Figure 1(b)), implying that the decreased NO production may not be due to the cytotoxic effect of PEF. These in vitro data suggested the anti-inflammatory potential of PEF. Hence, we focused on PEF in the subsequent cell and animal studies.

### 3.3. Effect of PEF on the Production of Inflammatory Mediators (NO and PGE_2_)

**Figure 2:**
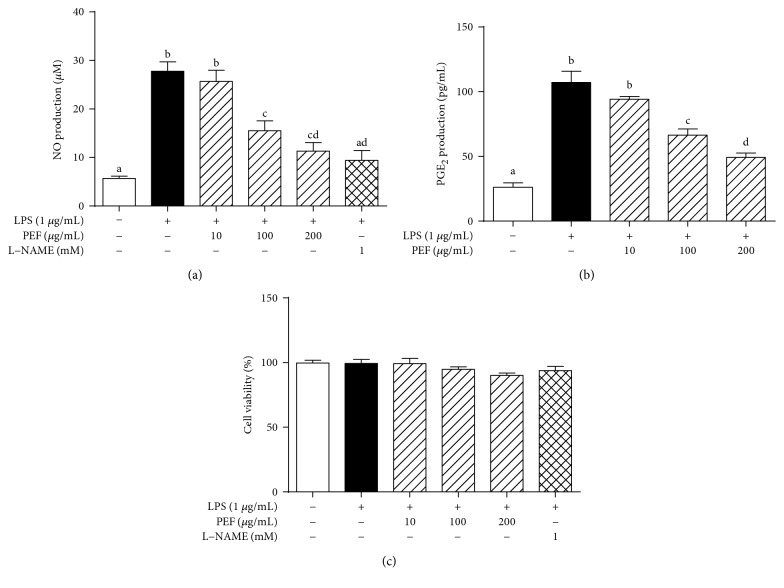
Effect of PEF on LPS-stimulated NO and PGE_2_ production in RAW 264.7 macrophages. RAW 264.7 macrophages were preincubated with 10–200 *μ*g/mL PEF for 2 h and then treated with 1 *μ*g/mL LPS for 24 h. (a) The nitrite concentration in the cultured medium was measured as an indicator of NO production by the Griess reaction. (b) PGE_2_ production in the cultured medium was determined by ELISA. (c) The cell viability was evaluated by MTT assay. L-NAME (1 mM) was used as a positive control. Results are shown as the mean ± SD (*n* = 3). The different letters represent the statistical differences at *p* < 0.05 among the groups by Tukey-Kramer's test.

The effect of PEF on the LPS-stimulated production of NO and PGE_2_ was further determined. As can be seen in Figures 2(a) and 2(b), PEF pretreatment prominently inhibited the production of NO and PGE_2_ induced by LPS treatment in a dose-dependent manner.  Figure 2(c) shows that PEF did not have any cytotoxicity at the concentrations of 10–200 *μ*g/mL. These results clearly demonstrated that the inhibitory effect of PEF on LPS-induced NO and PGE_2_ production may not be attributed to cytotoxic effects.

### 3.4. Effect of PEF on the Expression of iNOS and COX-2

**Figure 3:**
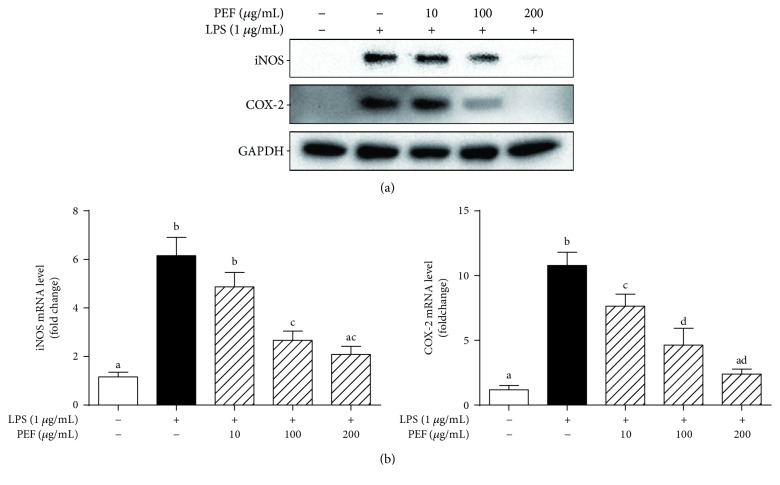
Effect of PEF on LPS-stimulated iNOS and COX-2 expression in RAW 264.7 macrophages. RAW 264.7 macrophages were preincubated with 10–200 *μ*g/mL PEF for 2 h and then treated with 1 *μ*g/mL LPS for 24 h. (a) The protein level of iNOS and COX-2 was determined by Western blot analysis. GAPDH was used as an endogenous control. (b) Total RNA was isolated and reverse-transcribed into cDNA for RT-PCR analysis of iNOS and COX-2 mRNA level. GAPDH was used as an endogenous control. Results are shown as the mean ± SD (*n* = 3). The different letters represent the statistical differences at *p* < 0.05 among the groups by Tukey-Kramer's test.

iNOS and COX-2 are two enzymes that play crucial roles in the synthesis of NO and PGE_2_ during inflammation, respectively [43]. Therefore, the protein expression of iNOS and COX-2 was determined in LPS-stimulated RAW 264.7 macrophages by Western blot analysis. As depicted in Figure 3(a), PEF pretreatment remarkably suppressed LPS-induced iNOS and COX-2 protein expression in a dose-dependent manner. Similar results were observed in the determination of mRNA levels of iNOS and COX-2 (Figure 3(b)). These results indicated that PEF-mediated inhibition of NO and PGE_2_ production was probably attributed to the downregulation of iNOS and COX-2 genes.

### 3.5. Effect of PEF on the Production of Proinflammatory Cytokines (TNF-*α*, IL-1*β*, and IL-6)

**Figure 4:**
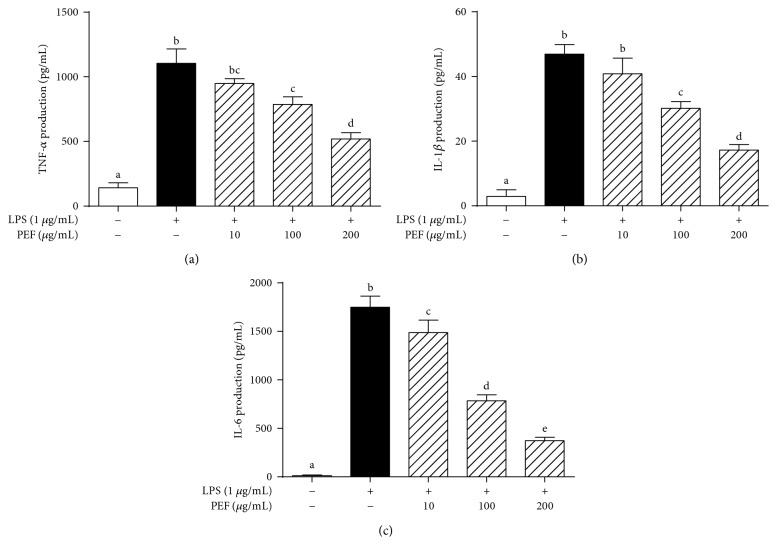
Effect of PEF on LPS-stimulated production of proinflammatory cytokines in RAW 264.7 macrophages. RAW 264.7 macrophages were preincubated with 10–200 *μ*g/mL PEF for 2 h and then treated with 1 *μ*g/mL LPS for 24 h. The concentrations of (a) TNF-*α*, (b) IL-1*β*, and (c) IL-6 in the cultured medium were determined by the commercial ELISA kits. Results are shown as mean ± SD (*n* = 3). The different letters represent the statistical differences at *p* < 0.05 among the groups by Tukey-Kramer's test.

Numerous studies have demonstrated that TNF-*α*, IL-1*β*, and IL-6, three well-known and early secreted proinflammatory cytokines, were involved in the acute phase response and systemic inflammation [4, 44]. According to the ELISAs presented in Figure 4, RAW 264.7 macrophages treated with LPS showed severe inflammatory responses, as manifested by significantly increased levels of those proinflammatory cytokines mentioned above. However, these inductions were efficiently reversed by PEF pretreatment in a dose-dependent manner. These data strongly supported the therapeutic potential of PEF in modulating inflammatory reactions.

### 3.6. Effect of PEF on the Activation of NF-*κ*B

**Figure 5:**
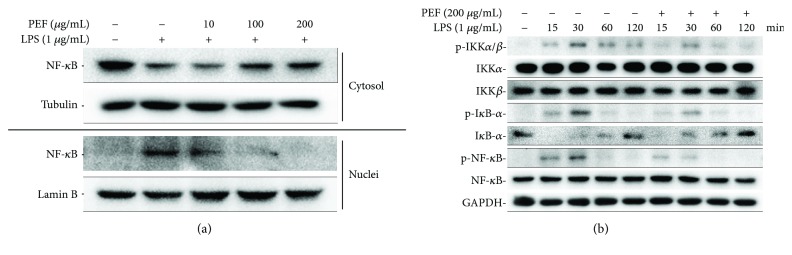
Effect of PEF on LPS-stimulated activation of NF-*κ*B in RAW 264.7 macrophages. (a) RAW 264.7 macrophages were preincubated with 10–200 *μ*g/mL PEF for 2 h and then treated with 1 *μ*g/mL LPS for 0.5 h. The nuclear and cytoplasmic extracts of cells were prepared, and the protein level of NF-*κ*B was determined by Western blot analysis. Lamin B and tubulin were used as endogenous controls for the nucleus and cytoplasm, respectively. (b) RAW 264.7 macrophages were preincubated with 200 *μ*g/mL PEF for 2 h; then the total protein was harvested at different time points (15, 30, 60, and 120 min) after stimulation with LPS (1 *μ*g/mL), and the protein levels of p-IKK*α*/*β*, IKK*α*, IKK*β*, p-I*κ*B-*α*, I*κ*B-*α*, p-NF-*κ*B, and NF-*κ*B were determined by Western blot analysis. GAPDH was used as an endogenous control.

Previous reports have concluded that NF-*κ*B is a critical transcription factor that regulates proinflammatory cytokine expression and mediates inflammatory responses [14, 45]. To assess whether the activation of NF-*κ*B was suppressed by PEF pretreatment in LPS-stimulated RAW 264.7 macrophages, the nuclear translocation of the NF-*κ*B/p65 subunit was examined by Western blot analysis. As shown in Figure 5(a), after stimulation with LPS alone, the amount of p65 in the nucleus increased significantly; however, the LPS-induced translocation of p65 from the cytosol to the nucleus was markedly inhibited by PEF pretreatment in a dose-dependent manner. To further assess the underlying molecular mechanisms, the inhibitory effect of PEF on LPS-stimulated phosphorylation of IKK*α*/*β*, NF-*κ*B, and I*κ*B-*α* as well as the degradation of I*κ*B-*α* was also investigated, which indicates the activation of NF-*κ*B. As expected, LPS-induced phosphorylation of these signaling molecules was obviously suppressed by PEF pretreatment. Moreover, LPS treatment caused prominent I*κ*B-*α* degradation as compared to the untreated control cells; nevertheless, PEF pretreatment notably recovered the protein level of I*κ*B-*α* (Figure 5(b)). These results suggested that the inhibitory effect of PEF on the production of inflammatory mediators and proinflammatory cytokines was attributed to the inactivation of NF-*κ*B in LPS-stimulated RAW 264.7 macrophages.

### 3.7. Effect of PEF on the Phosphorylation of MAPKs

**Figure 6:**
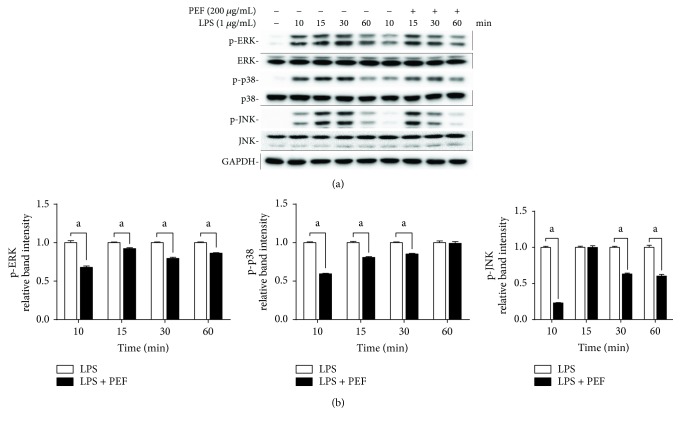
Effect of PEF on LPS-stimulated phosphorylation of MAPKs in RAW 264.7 macrophages. (a) RAW 264.7 macrophages were preincubated with 200 *μ*g/mL PEF for 2 h, and then, the total protein was harvested at different time points (10, 15, 30, and 60 min) after stimulation with LPS (1 *μ*g/mL), and the protein levels of p-ERK, ERK, p-p38, p38, p-JNK, and JNK were determined by Western blot analysis. GAPDH was used as an endogenous control. (b) Relative intensity of the immunoreactive bands was analyzed, and results are shown as the mean ± SD (*n* = 3). Statistical difference between two groups in the presence and absence of PEF was evaluated by Student's *t*-test. ^a^ *p* < 0.05, compared to the LPS-treated group.

It is well established that the phosphorylation of MAPKs plays significant roles in the activation of NF-*κ*B in LPS-stimulated macrophages [46, 47]. To gain a better understanding of the underlying mechanisms, the phosphorylation of three MAPKs including extracellular signal-regulated kinases (ERK1/2), c-Jun N-terminal kinase (JNK), and p38 was examined by Western blot analysis. As presented in Figure 6, LPS-induced phosphorylation of ERK, JNK, and p38 was effectively suppressed by PEF pretreatment, indicating that PEF exerted an anti-inflammatory effect partially through attenuating the phosphorylation of MAPKs stimulated by LPS treatment.

### 3.8. Effect of PEF on APAP-Induced Liver Injury

**Figure 7:**
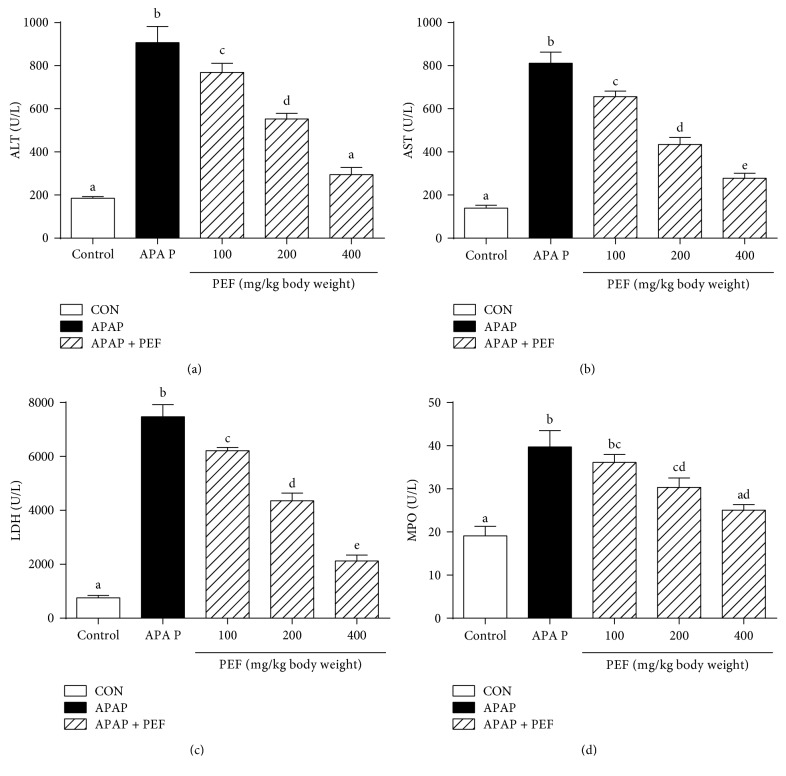
Effect of PEF on APAP-induced liver injury. Mice were intragastrically administered with either PBS or PEF (100, 200, and 400 mg/kg body weight) once daily for 7 consecutive days prior to a single administration of APAP (500 mg/kg body weight). Mice were killed at 6 h after APAP challenge. Serum activities of (a) ALT, (b) AST, (c) LDH, and (d) MPO were determined by the commercial kits. Results are shown as the mean ± SD (*n* = 8). The different letters represent the statistical differences at *p* < 0.05 among the groups by Tukey-Kramer'stest.

**Figure 8:**
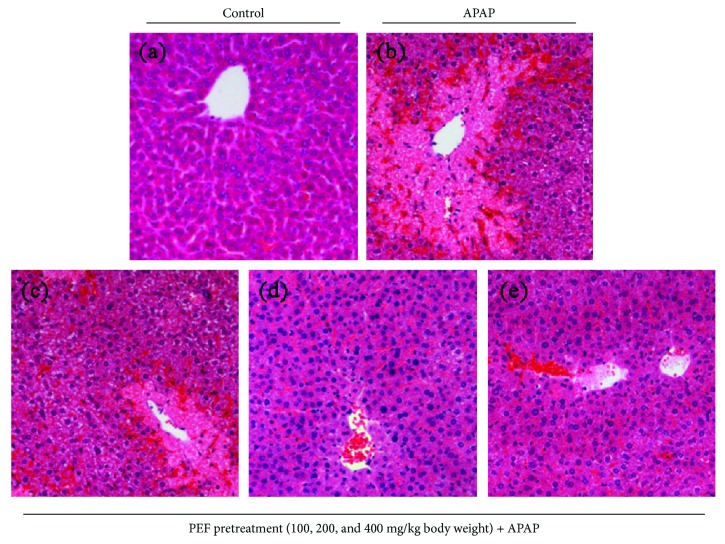
Effect of PEF on histological changes in APAP-intoxicated mouse livers. Liver sections were stained with hematoxylin and eosin (original magnification of 100x). (a) Control group. (b) APAP-intoxicated group. (c) PEF (100 mg/kg body weight) + APAP. (d) PEF (200 mg/kg body weight) + APAP. (e) PEF (400 mg/kg body weight) + APAP.

It was reported that the administration of an excessive dose of APAP to mice could lead to severe hepatocyte necrosis and acute liver injury. APAP-induced early hepatocyte necrosis activated the innate immune cells and caused hepatic inflammation, which in turn influenced the severity of APAP-induced hepatotoxicity [48]. In view of the anti-inflammatory effect of PEF observed in the previous experiments, we further investigated the hepatoprotective effect of PEF on APAP-induced liver injury. As presented in Figure 7, mice treated with APAP (500 mg/kg body weight) showed a severe hepatotoxic effect with prominent evidence of increased serum activities of ALT and LDH, which was in agreement with earlier studies [7, 22]. However, PEF pretreatment (100, 200, and 400 mg/kg body weight) for 7 consecutive days prior to APAP administration showed a dose-dependently reductive effect towards these APAP-caused increases. A similar result was obtained from the determination of MPO activity, another important indicator which reflects the degree of damage and inflammation [49]. Further histopathological analysis provided supportive evidence for the biochemical parameters assay (Figure 8). A normal tissue structure with a typical hepatic cellular architecture was presented in the untreated control group. The sections of liver samples taken from APAP-intoxicated mice revealed extensive histological changes, as characterized by severe hepatocellular degeneration and necrosis, cytoplasmic vacuolation, and inflammatory cell infiltration. Interestingly, PEF pretreatment significantly ameliorated these APAP-induced injuries in the livers of mice. These data suggested that PEF protected against APAP-induced acute hepatic damage.

### 3.9. Effect of PEF on APAP-Induced Hepatic Inflammation

**Figure 9:**
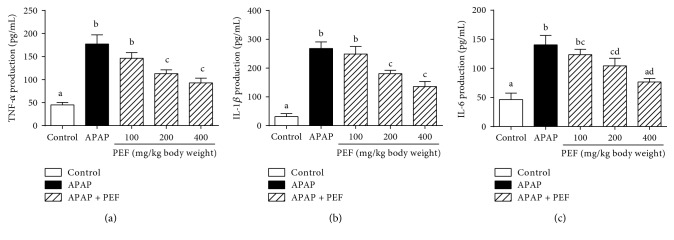
Effect of PEF on proinflammatory cytokines production in APAP-intoxicated mice. Mice were intragastrically administered with either PBS or PEF (100, 200, and 400 mg/kg body weight) once daily for 7 consecutive days prior to a single administration of APAP (500 mg/kg body weight). Mice were killed at 6 h after APAP challenge. Serum concentrations of (a) TNF-*α*, (b) IL-1*β*, and (c) IL-6 were determined by the commercial ELISA kits. Results are shown as the mean ± SD (*n* = 8). The different letters represent the statistical differences at *p* < 0.05 among the groups by Tukey-Kramer's test.

To evaluate the effect of PEF on APAP-induced hepatic inflammation, serum concentrations and mRNA levels of several proinflammatory factors were investigated. According to the ELISAs and RT-PCR analysis, APAP administration significantly increased levels of TNF-*α*, IL-1*β*, and IL-6 compared to the untreated control group, indicating severe inflammatory responses in APAP-intoxicated mouse livers. However, these aberrant increases were notably suppressed by PEF pretreatment (Figure 9 and Figure S1). The mRNA levels of monocyte chemotactic protein 1 (MCP-1) and macrophage inflammatory protein-1*α* (MIP-1*α*), two generalized proinflammatory chemokines which actively participate in the inflammatory responses and attract immune cells to the site of inflammation, were also determined, and the results of which presented an extreme consistency (Figure S1). These results indicated that PEF alleviated APAP-induced liver injury by inhibiting the expression of proinflammatory factors.

### 3.10. Effect of PEF on APAP-Induced Activation of TLRs, MAPKs, and NF-*κ*B

**Figure 10:**
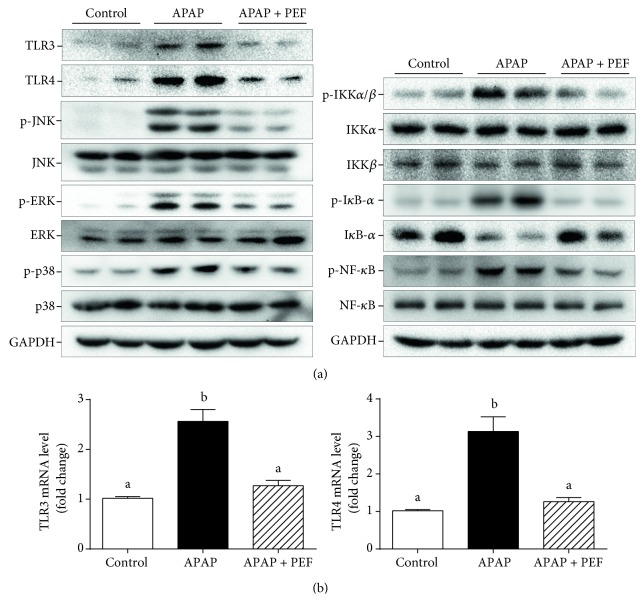
Effect of PEF on the activation of TLRs, MAPKs, and NF-*κ*B in APAP-intoxicated mouse livers. (a) Total protein was extracted from liver tissues, and the protein levels of TLR3, TLR4, p-JNK, JNK, p-ERK, ERK, p-p38, p-38, p-IKK*α*/*β*, IKK*α*/*β*, p-I*κ*B-*α*, I*κ*B-*α*, p-NF-*κ*B, and NF-*κ*B were determined by Western blot analysis. GAPDH was used as an endogenous control. (b) Total RNA was isolated from liver tissues and reverse-transcribed into cDNA for RT-PCR analysis of TLR3 and TLR4 mRNA level. GAPDH was used as an endogenous control. Results are shown as the mean ± SD (*n* = 8). The different letters represent the statistical differences at *p* < 0.05 among the groups by Tukey-Kramer's test.

Accumulating evidence has demonstrated the increased expression of TLRs in APAP or CCl_4_-intoxicated mouse livers [22, 23]. As reported previously, APAP administration obviously induced the expression levels of TLR3 and TLR4 compared to the untreated control group, whereas PEF pretreatment noticeably inhibited these elevations (Figures 10(a) and 10(b)), implying the downregulated NF-*κ*B-mediated inflammatory cascade. Therefore, the activation status of MAPKs and NF-*κ*B was further investigated. As expected, the phosphorylation of MAPKs and the activation of NF-*κ*B were dramatically inhibited by PEF pretreatment (Figure 10(a)), and the results of which were closely parallel with those observed in previous LPS-stimulated RAW 264.7 macrophages. Taken together, these in vivo results indicated that PEF protected against APAP-induced hepatotoxicity and hepatic inflammation possibly through the inactivation of the TLR/MAPK/NF-*κ*B signaling pathway.

### 3.11. Identification and Quantitative Determination of Main Components in PEF

**Figure 11:**
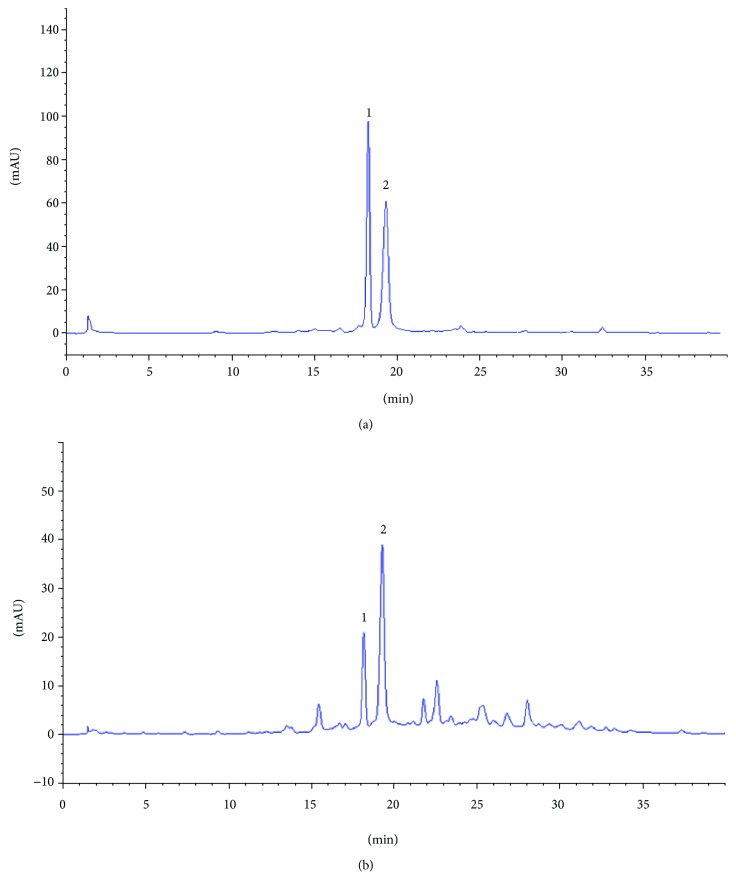
RP-HPLC profiles of (a) the polyphenolic standards and (b) the polyphenolic compounds in PEF at 280 nm. Peaks: 1, corilagin; 2, geraniin.

To better understand the major constituents related to the activity of PEF, the main polyphenolic compounds, 1 and 2, were purified by ODS column chromatography and semipreparative reversed-phase HPLC. Identifications of these two purified compounds were achieved by spectroscopic techniques and comparing the spectroscopic data with previously published data [50, 51]. Their structures were identified as corilagin (1) and geraniin (2). At last, 1 and 2 were quantified by HPLC analysis. Chromatograms of standards and PEF solution are shown in Figure 11, and quantitative data are shown in Table S1, which indicated that geraniin (286.1 *μ*g/mg) was the major polyphenolic compound in PEF, followed by corilagin (54.2 *μ*g/mg).

## 4. Discussion and Conclusions

Naturally occurring polyphenolic compounds, which are abundantly presented in fruits, vegetables, and cereals, have attracted widespread attention due to their medicinal properties [25, 28, 29]. Through literature search, we find that there are few reports connecting the anti-inflammatory and hepatoprotective effects to the polyphenolic constituents of *A. wilkesiana* and revealing the potential molecular mechanisms. In this study, a polyphenol-enriched fraction (PEF), fractionated from the decoction of *A. wilkesiana* by column chromatography with Diaion HP-20 resin, was found to effectively inhibit LPS-stimulated inflammatory responses in RAW 264.7 macrophages as well as hepatic inflammation in APAP-induced liver injury mice via the suppression of the TLR/MAPK/NF-*κ*B signaling pathway. Additionally, we found that geraniin and corilagin, two main polyphenolic compounds present in PEF, are responsible, at least to some extent, for PEF's anti-inflammatory effect. Our findings provide a better understanding of the positive relationship between total phenolic or flavonoid content in *A. wilkesiana* and its anti-inflammatory and hepatoprotective activities.

LPS-stimulated RAW 264.7 macrophages produce excessive inflammatory mediators and proinflammatory cytokines and act as an experimentally convenient model to evaluate the anti-inflammatory activity of drug candidates [3, 4, 44]. In the present study, we found that the pretreatment of RAW 264.7 macrophages with PEF (200 *μ*g/mL) significantly inhibited NO and PGE_2_ production by the downregulation of iNOS and COX-2 genes at the transcriptional level (Figures 2 and 3). Additionally, PEF was also shown to suppress LPS-induced TNF-*α*, IL-1*β*, and IL-6 production in RAW 264.7 macrophages (Figure 4). These data indicated the anti-inflammatory effect of PEF and its therapeutic potential for inflammatory diseases. The inhibitory effect of PEF on APAP-induced liver injury and hepatic inflammation provided supportive evidence for the hypothesis. It is well-known that the increased activities of ALT, AST, and LDH enzymes are diagnostic indicators of hepatocellular damage [45, 52]. In agreement with earlier studies, serum activities of ALT, AST, and LDH were significantly increased after APAP administration, whereas these elevations were decreased by PEF pretreatment (100–400 mg/kg body weight) (Figure 7), suggesting that PEF may protect hepatocytes against the toxic effects of APAP. Histopathological analysis provided visual evidence for the hepatoprotective effect of PEF, as manifested by the restoration toward histomorphological variations (Figure 8). Despite a wide recognition that the toxicity of drug metabolism in term of oxidative stress plays a critical role in APAP-induced liver injury, there is an increasing awareness that infiltrating inflammatory cells and inflammation may also be implicated in the pathogenesis [17, 18, 53–55]. Accumulating evidence has demonstrated that acute APAP intoxication increased circulation levels of many proinflammatory cytokines [56–58]. In our study, APAP administration induced prominent production of several proinflammatory factors at both plasma and mRNA levels; however, PEF pretreatment distinctly reversed these changes (Figure 9 and Figure S1). It should also be noted that MPO is another biomarker indicating the infiltration of inflammatory cells, especially neutrophils into tissues, and commonly regarded as an acute response to liver injury, hepatic stress, or unknown inflammatory stimuli [49, 59]. Here, APAP administration led to a prominent increase in MPO activity; nevertheless, PEF pretreatment could markedly dispute this increase (Figure 7). On this basis, it is suggested that PEF could attenuate APAP-induced hepatic inflammation and the consequent acute liver injury.

NF-*κ*B is known to be a key transcription factor involved in cellular inflammatory responses. In the unstimulated condition, NF-*κ*B is sequestered in the cytoplasm in an inactivated form by interaction with I*κ*B-*α*. When stimulated by extracellular stimuli, activated IKKs cause the phosphorylation and subsequent degradation of I*κ*B-*α* and then promote the nuclear translocation of NF-*κ*B, ultimately leading to the transcription of proinflammatory genes [10–12]. In this work, PEF pretreatment could effectively restrain LPS-induced nuclear translocation of the NF-*κ*B/p65 subunit (Figure 5(a)), indicating the inactivation of NF-*κ*B. Moreover, Western blot analysis revealed that the phosphorylation of IKK*α*/*β*, I*κ*B-*α*, and NF-*κ*B as well as the degradation of I*κ*B-*α* induced by LPS or APAP was also reversed by PEF pretreatment (Figures 5(b) and 10(b)), supporting the inactivation of NF-*κ*B by PEF. From these data, it is likely that PEF-mediated suppression of LPS or APAP-induced inflammatory mediators and proinflammatory factor expression is mainly attributed to the inhibitory action of PEF on the NF-*κ*B pathway.

NF-*κ*B activation is also regulated by various cellular kinases, especially MAPKs, a family of serine/threonine kinases mainly including JNK, ERK1/2, and p38. Accumulating evidence has demonstrated that activated MAPKs are involved in the production of inflammatory mediators and proinflammatory cytokines as well as the expression of iNOS and COX-2 through NF-*κ*B activation in LPS-stimulated RAW 264.7 macrophages and drug-intoxicated livers [14, 42, 44, 60]. Therefore, the activation of MAPKs is another important molecular mechanism to evaluate the inflammatory responses. Our results showed that the phosphorylation of ERK1/2, JNK, and p38 induced by LPS or APAP was obviously inhibited by PEF pretreatment (Figures 6(a) and 10(a)). These findings suggested that the suppression of MAPKs by PEF may contribute to PEF-mediated inhibition of the NF-*κ*B pathway.

Previous studies have demonstrated that TLRs act as crucial regulators in the recognition of exogenous pathogen-associated molecular patterns (PAMPs) and DAMPs released from stressed or dying cells during tissue damage [18, 61–63]. Several reports have revealed the involvement of activated TLR3 or TLR4 in APAP-intoxicated mouse livers. TLR3 activation enhanced the expression of phosphorylated JNK and contributed to APAP-induced liver injury [48]. TLR4-KO mice were reported to be relatively protected from APAP-caused liver necrosis and organ dysfunction [22]. Our data showed that APAP administration caused increased expression of TLR3 and TLR4, which in turn promoted the activation of MAPKs and NF-*κ*B and the subsequent production of inflammatory mediators and proinflammatory factors, ultimately leading to the aggravated liver injury. However, all the changes were significantly restored by PEF pretreatment (Figure 10), illustrating that the hepatoprotective effect of PEF on APAP-induced acute liver injury may be achieved by its ability to mediate the TLR/MAPK/NF-*κ*B signaling pathway.

Chemical investigation showed that PEF was a rich source of polyphenolic compounds (Table 1), especially geraniin and corilagin (Figure 11 and Table S1), which have been proved to exhibit hepatoprotective and anti-inflammatory activities. Geraniin could protect mice against CCl_4_-induced hepatotoxicity and inhibit LPS-induced expression of inflammatory mediators and proinflammatory cytokines by suppressing the Akt-mediated NF-*κ*B pathway [64, 65]. Corilagin was protective against GalN/LPS-induced liver injury by attenuating oxidative stress and apoptosis [66]. It was shown that corilagin could inhibit schistosomiasis-induced hepatic fibrosis via the miR21/smad7/ERK pathway [67]. A recent study has also reported that corilagin exerted an anti-inflammatory effect by downregulating the TLR4 signaling molecules to ameliorate the extreme inflammatory status in sepsis [68]. Therefore, the presence of geraniin and corilagin and other polyphenolic constituents in PEF may be the main contributing factor responsible for its anti-inflammatory and hepatoprotective activities.

In conclusion, the results of the current study clearly demonstrate that PEF exerts anti-inflammatory effects on LPS-stimulated RAW 264.7 macrophages and APAP-induced hepatotoxicity in mice mainly through the inactivation of the NF-*κ*B pathway via a blockade of TLR and MAPK activation. Furthermore, the anti-inflammatory activity of PEF was found to be highly associated with its polyphenolic constituents. These findings provide the scientific evidence for further application of the polyphenol-enriched fraction from *A. wilkesiana* in therapeutic agents for inflammatory diseases.

## Figures and Tables

**Table 1:** Contents of total phenolics and total flavonoids of different *A. wilkesiana* extracts

Extract	Total phenolics (*μ*g GAE/mg extract)	Total flavonoids (*μ*g RE/mg extract)
WE	261.1 ± 2.5	152.6 ± 3.8
WF	87 ± 3.1	38 ± 3.5
PEF	661.4 ± 5.2^a^	306.5 ± 7.5^a^

Results are shown as the mean ± SD (*n* = 3). ^a^ *p* < 0.05, compared to the other extracts.

## Data Availability

The data used to support the findings of this study are available from the corresponding author upon request.

## Supplementary Materials

Supplementary 1 Figure S1: effect of PEF on mRNA levels of proinflammatory factors in APAP-intoxicated mice. Mice were intragastrically administered with either PBS or PEF (100, 200, and 400 mg/kg body weight) once daily for 7 consecutive days prior to a single administration of APAP (500 mg/kg body weight). Mice were killed at 6 h after APAP challenge. Total RNA from liver tissues was isolated and reverse-transcribed into cDNA for RT-PCR analysis of (a) TNF-*α*, (b) IL-1*β*, (c) IL-6, (d) MCP-1, and (e) MIP-1*α* mRNA level. GAPDH was used as an endogenous control. Results are shown as the mean ± SD (*n* = 8). The different letters represent the statistical differences at *p* < 0.05 among the groups by Tukey-Kramer's test.

Supplementary 2 Table S1: calibration curves and contents of the polyphenolic compounds in PEF from *A. wilkesiana*.
