# Supplementary figures and images for: Anti-Inflammatory Effect of a Polyphenol-Enriched Fraction from Acalypha wilkesiana on Lipopolysaccharide-Stimulated RAW 264.7 Macrophages and Acetaminophen-Induced Liver Injury in Mice
Source: Oxid Med Cell Longev. 2018 Aug 7;2018:7858094. doi: 10.1155/2018/7858094 (PMC6109486; doi:10.1155/2018/7858094)

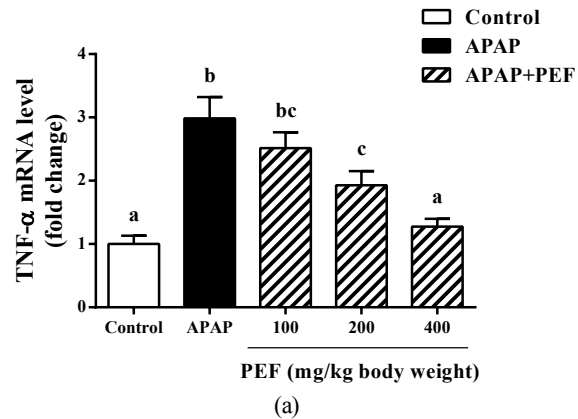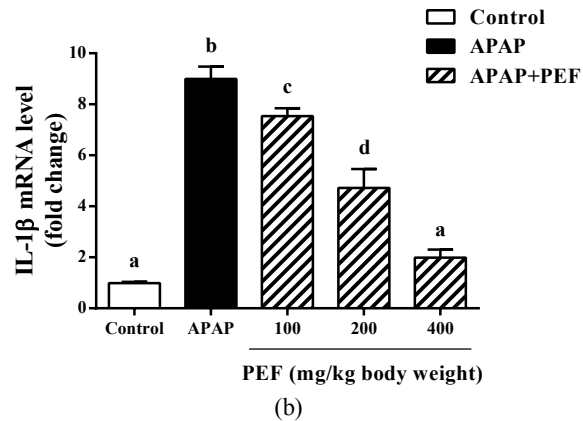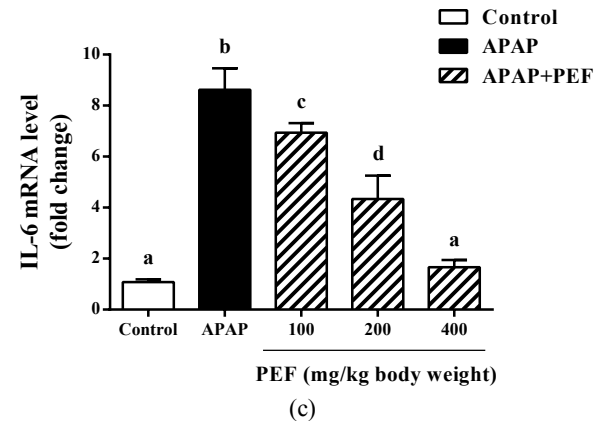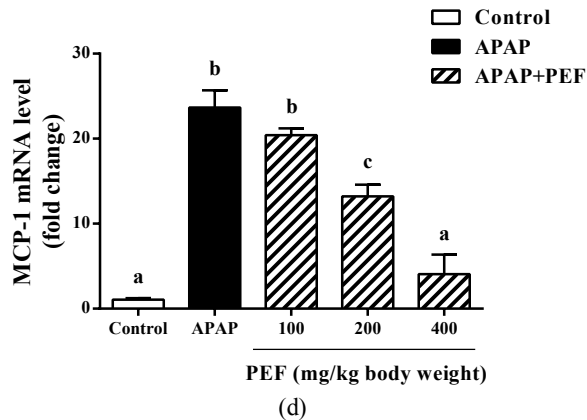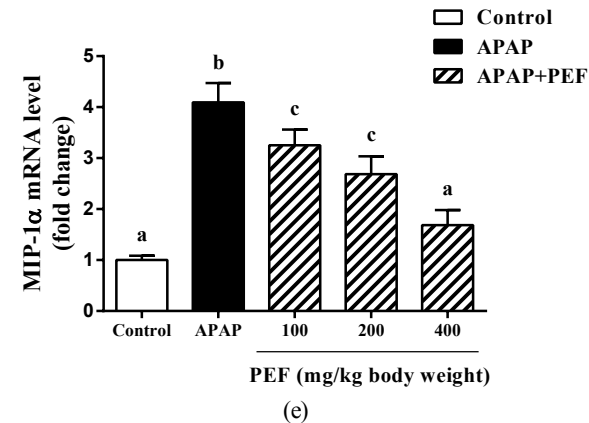

Supplement: Supplementary 1 — Figure S1: effect of PEF on mRNA levels of proinflammatory factors in APAP-intoxicated mice. Mice were intragastrically administered with either PBS or PEF (100, 200, and 400 mg/kg body weight) once daily for 7 consecutive days prior to a single administration of APAP (500 mg/kg body weight). Mice were killed at 6 h after APAP challenge. Total RNA from liver tissues was isolated and reverse-transcribed into cDNA for RT-PCR analysis of (a) TNF-α, (b) IL-1β, (c) IL-6, (d) MCP-1, and (e) MIP-1α mRNA level. GAPDH was used as an endogenous control. Results are shown as the mean ± SD (n = 8). The different letters represent the statistical differences at p < 0.05 among the groups by Tukey-Kramer's test. [file 7858094.f1.pdf]
