# Supplementary material for: Anti-Inflammatory Effect of a Polyphenol-Enriched Fraction from Acalypha wilkesiana on Lipopolysaccharide-Stimulated RAW 264.7 Macrophages and Acetaminophen-Induced Liver Injury in Mice
Source: Oxid Med Cell Longev. 2018 Aug 7;2018:7858094. doi: 10.1155/2018/7858094 (PMC6109486; doi:10.1155/2018/7858094)
Supplement: Supplementary 2 — Table S1: calibration curves and contents of the polyphenolic compounds in PEF from A. wilkesiana. [file 7858094.f2.doc]

| **TABLE S1:Calibration curves and contents of the polyphenolic compounds in PEF from *A.wilkesiana*** | | | | | |
| --- | --- | --- | --- | --- | --- |
| No | polyphenolic compound | content  (μg/mg) | t_R_ (min) | equation of regression  (Y = aX + b) | R^2^ |
| **1** | corilagin | 54.2 | 18.5 | Y = 1169X - 7.639 | 0.9992 |
| **2** | geraniin | 286.1 | 19.7 | Y = 603.97X - 81.48 | 0.9994 |
